# Pleural decompression procedural safety for traumatic pneumothorax and haemothorax: Kelly clamps *versus* fine artery forceps

**DOI:** 10.1111/1742-6723.14019

**Published:** 2022-05-26

**Authors:** Mark Fitzgerald, Thomas Allen, Shifeng Bai, Biswadev Mitra, Wing Chiu, Dries Helsloot, Chris Groombridge, Joseph Mathew, Yesul Kim

**Affiliations:** ^1^ National Trauma Research Institute Monash University Melbourne Victoria Australia; ^2^ Trauma Service Alfred Health Melbourne Victoria Australia; ^3^ Department of Mechanical and Aerospace Engineering Monash University Melbourne Victoria Australia; ^4^ Emergency and Trauma Centre Alfred Health Melbourne Victoria Australia; ^5^ Emergency Medicine AZ Groeninge Kortrijk Belgium

**Keywords:** forceps, pleural puncture force, pneumothorax

## Abstract

**Objective:**

The present study aimed to determine the difference in force required to puncture simulated pleura comparing Kelly clamps to fine artery forceps. The treatment of symptomatic traumatic pneumothorax and haemothorax involves puncture of the parietal pleura to allow decompression. This is usually performed using Kelly clamps or fine artery forceps. Over‐puncture pulmonary injury risk increases with the force used.

**Methods:**

An experienced single operator performed puncturing of simulated parietal pleura on a thoracic mannequin while wearing a force sensor under gloves. The force imparted at the device tip onto the parietal pleura was estimated by subtracting the force required to hold the device from the total force. Outcome variables were the total maximum force and force imparted by the device.

**Results:**

There were 11 simulated procedures completed, seven using Kelly clamps and four using fine artery forceps. After subtracting the force required to hold the chosen forceps, the median value of pleural puncture force using Kelly clamps was 52.91 N (IQR 36.68–63.56) and 10.70 N (IQR 7.64–26.56) using fine artery forceps (*P* = 0.006).

**Conclusion:**

A significantly increased force was required to puncture simulated parietal pleura using Kelly clamps compared to fine artery forceps. This higher puncture force will be associated with increased instrument acceleration at the time of pleural puncture, which may result in an increased risk of injury to the underlying lung. Based on these data, clinicians may reduce the risk of pulmonary injury by using fine artery forceps rather than Kelly clamps when performing pleural decompression.

## Graphical abstract

The treatment of symptomatic traumatic pneumothorax and haemothorax involves puncture of the parietal pleura to allow decompression. The present study demonstrated that a significantly increased force was required to puncture simulated parietal pleura using Kelly clamps compared to fine artery forceps. This higher puncture force will be associated with increased instrument acceleration at the time of pleural puncture, which may result in an increased risk of injury to the underlying lung.

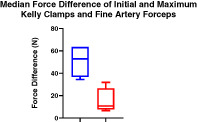

**Key findings**

- Over‐puncture injury is a risk of pleural decompression.
- The risk increases with the force used.
- The force used is proportional to the tip surface of the instrument.

## Introduction

The treatment of symptomatic traumatic pneumothorax and haemothorax involves puncture of the parietal pleura to allow decompression. This is followed by tube thoracostomy to drain air or fluid in the inter‐pleural space.

‘Over‐puncture’ injuries are well recognised in medicine,^1^ and iatrogenic injury to underlying organs has been reported as a potential complication of pleural decompression. The underlying lung is the organ that most commonly may sustain an iatrogenic injury, while case reports of injuries to the oesophagus, stomach, bowel, liver, spleen, diaphragm, heart and major blood vessels have also been identified as complications of pleural decompression and tube thoracostomy.^2^, ^3^

**Figure 1:**
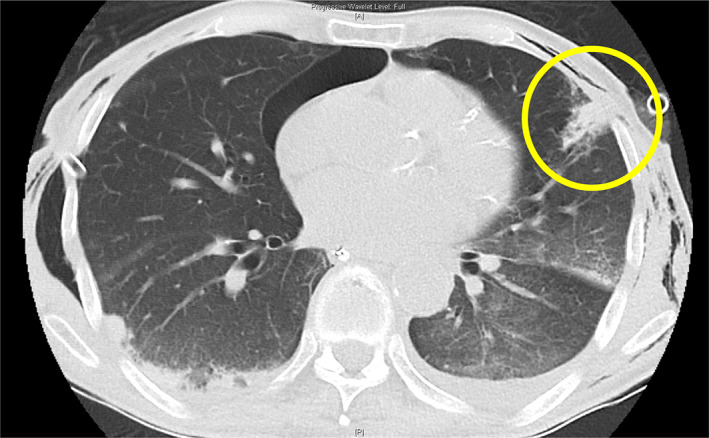
CT scan demonstrating left lung injury (circled) following Kelly clamp pleural puncture.

Puncture of the parietal pleural is usually performed using Kelly clamps^3^ or fine artery forceps.^4^ The puncture resistance will depend on the nature of puncture attempt, with the two most important features being point sharpness and force.^1^ Kelly clamps are longer when compared to fine artery forceps. In addition, the tips of the Kelly clamps have a 2.7 times larger surface area than fine artery forceps. Based on the formula Pressure = Force/Area, and that a larger surface area will require a greater force for the same pressure to be applied, it was hypothesised that puncturing the pleura with Kelly clamps would require anywhere from two to three times as much force as compared to using fine artery forceps, increasing the risk of underlying pulmonary injury (Fig. 1). The aim of the present study was to compare the total force and force imparted to the tip of the instrument when using Kelly clamps *versus* artery forceps for parietal pleural puncture.

## Methods

**Figure 2:**
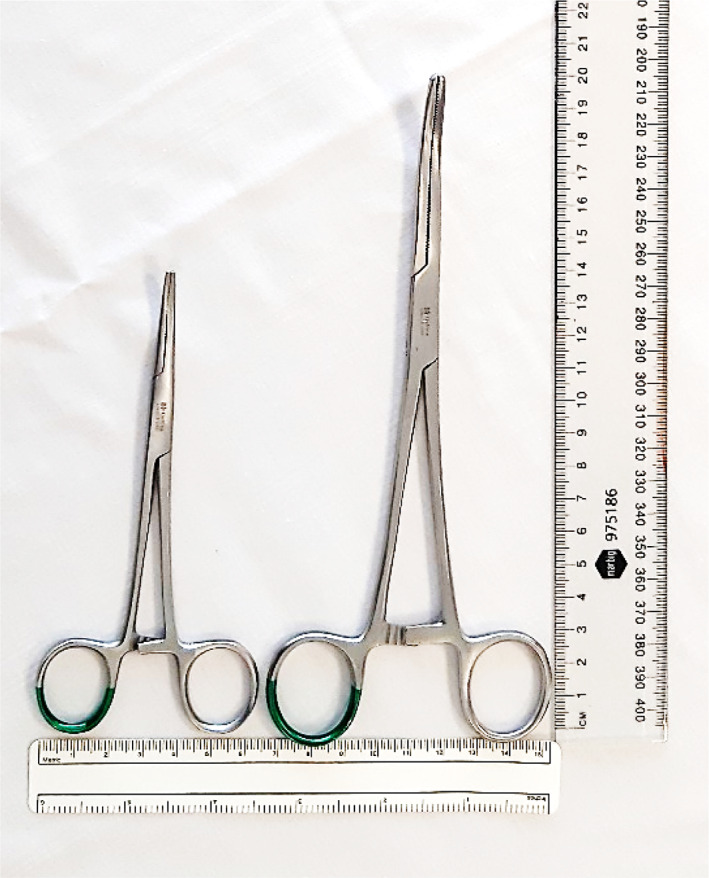
Fine artery forceps (left) and Kelly clamps (right).

Fine artery forceps and Kelly clamps were compared in the present study. The fine artery forceps used measured 13.5 cm in length and 0.03 cm^2^ in tip surface area. The Kelly clamps used measured 20 cm in length and had a tip surface area of 0.08 cm^2^ (Fig. 2).

**Figure 3:**
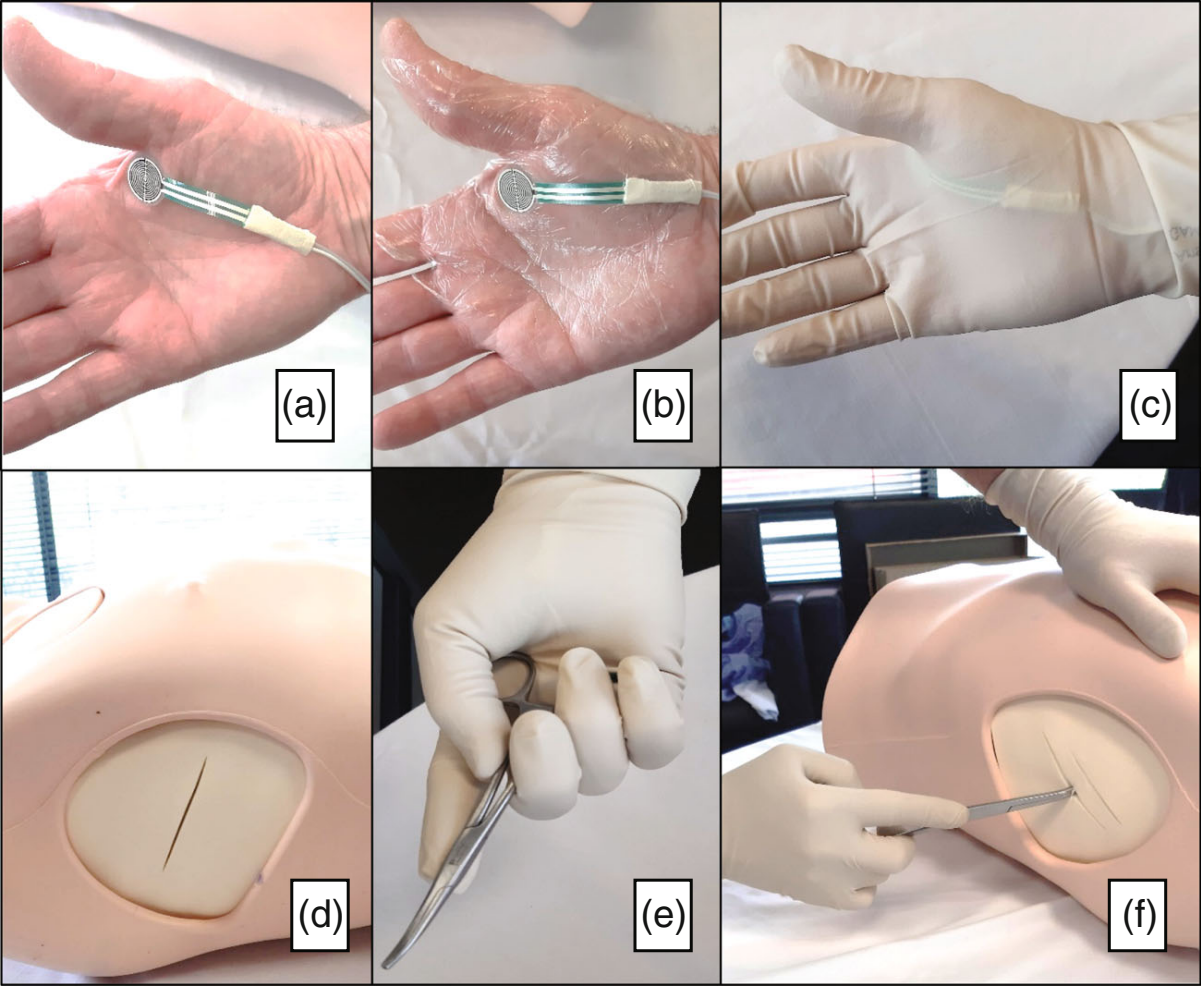
(a) Pressure sensor positioning; (b) Tegaderm™ application; (c) post gloving; (d) standardised incision; (e) fine artery forceps hold; (f) Kelly clamp hold.

Simulated procedures were performed on a thoracic mannequin (Chest Drain & Needle Decompression Trainer; Limbs and Things Australia Pty Ltd, Melbourne, Australia) used in the Royal Australasian College of Surgeons Early Management of Severe Trauma Course (EMST or ATLS) (Fig. 3). To allow for the replication of a chest drain insertion, the trainer was fitted with removable axillary pads. These pads are designed to mimic both skin as well as the parietal pleura. For the present study, the trainer was fitted with the Standard Chest Drain Pads.

A force measurement system was developed using a proprietary Arduino^©^ Motherboard and circuitry attached to a pressure sensor of 18.3 mm diameter and HC‐05 Bluetooth receiver (Fig. 3a). A Python code (Python Software Foundation, Wilmington, DE, USA) for PC was developed to allow this module to transmit and save data in a Microsoft® excel spreadsheet. The resistance of the sensor with no applied pressure was 3 000 000 Ω. This was connected in series to a resistor with a fixed resistance of 1000 Ω. The system measured a change in resistance caused by pressure on the pressure sensor. The greater the pressure, the lower the variable resistance became. Higher resistance decreased the voltage at the fixed resistor and this voltage reading was measured.

The sensor data were transferred via the Bluetooth module to the connected COM port and the Python programme at a rate of 5 readings per second. These data were then collated into an excel spreadsheet. The sensor was attached to the thenar eminence of the palm of an experienced proceduralist (Fig. 3a). The sensor was then secured with Tegaderm™, to reduce any potential movement of the sensor during the procedure (Fig. 3b). The same experienced proceduralist performed all the procedures to reduce inter‐proceduralist variability. Double gloves were worn to mimic consistency between the simulated chest drain procedures and those performed on live patients (Fig. 3c).

An incision was first made into the chest trainer's skin tissue using a surgical scalpel. This was to ensure that the choice of forceps did not influence the depth of the incision made (Fig. 3d). A typed slip of paper denoting ‘fine artery forceps’ or ‘Kelly clamps’ was then chosen blindly 12 times from a pool of 20 and not replaced. The sample size required was four for each procedure. All insertion data were collected until a minimum of four in each group was reached. The chosen forceps were held with the finger ring of the forceps being held against the underlying sensor on the thenar eminence (Fig. 3e,f). The forceps were then placed in the previously made skin incision against the mannequin's parietal pleura.

At this point in time, there was no force being used against the simulated chest wall or pleura by the forceps. The Python programme was then run, and the forceps were inserted puncturing the parietal pleura of the mannequin, with this force being measured. The procedure was performed using a one‐handed method, with the other hand being placed away from the procedure. This was to ensure that the only force used on the forceps was from the hand with the sensor attached. Also, the authors teach and practice a one‐handed technique for pleural puncture, and this technique reflected that practice. The pads in the chest decompression trainer were replaced to ensure that any new procedure performed was not influenced by previous procedures.

**Figure 4:**
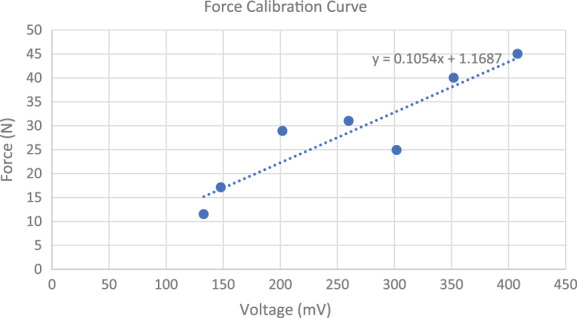
Force calibration curve showing the maximum force gauge reading and the associated maximum voltage reading from the force sensor module. Line of best fit shown *N* = 0.1054 mV + 1.1687.

The force measurement system recorded data as a voltage (mV), requiring conversion to force (N). The system was calibrated using a force gauge and a line of best fit, facilitating conversion of data from voltage to force (Fig. 4). A one‐tailed Mann–Whitney test was performed to compare the absolute maximum forces achieved using either instrument. A two‐tailed Mann–Whitney test was performed when comparing the absolute maximum forces applied. The median difference between the instrument holding force and the maximum pleural puncture force was compared between the two sets of forceps using a two tailed Mann–Whitney test.

Force curves were derived by defining the maximum force recorded as the synthetic pleural puncture force in each individual procedure. The time at which the pleura was punctured was defined as Time = 0.0 s. The median values, with IQR, at each time point were plotted (Fig. 4). All data analysis was performed in GraphPad Prism 8 (GraphPad Software, San Diego, CA, USA). A *P*‐value <0.05 was defined to be statistically significant. Assuming a force of 50 N would be imparted to the parietal pleura by Kelly forceps, to assess for a 50% reduction in force using 90% and 95% confidence intervals, a total of eight procedures were required, four with each device.

## Results

The relationship between the voltage readings of the device and the force applied was determined to be *N* = 0.1054 mV + 1.1687. This result was then applied to all data. Results from 11 procedures, seven using the Kelly clamps, and four using the fine artery forceps were analysed. A fifth fine artery forceps procedure was performed but failed to yield results because of a motherboard misconnection.

**Figure 5:**
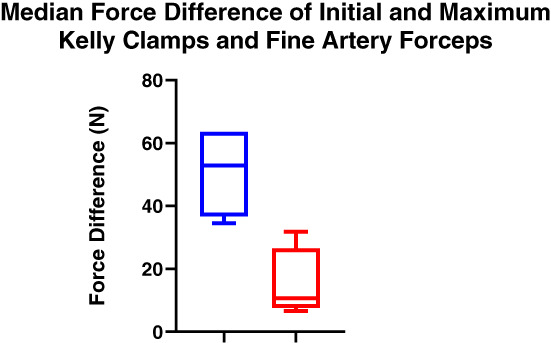
Box plot comparing the peak force values (*N*) recorded during each pleural puncture using Kelly clamps (*n* = 7) (blue) and fine artery forceps (*n* = 4) (red).

Figure 5 demonstrates the median difference between the maximum force of each procedure. The median difference shown using the Kelly clamps was 52.91 N (*n* = 7), and 10.70 N using the fine artery forceps (*n* = 4). The difference between these values was significant (*P* = 0.0061).

The Kelly clamps and fine artery forceps were held single‐handed, and when the force curves of the two sets of forceps were compared graphically, the Kelly clamps had a wider range of values at nearly all time points prior to the puncturing of the pleura and at the time of simulated pleural puncture.

## Discussion

Medical staff have a variable exposure to emergency pleural decompression in trauma with potentially higher complication rates as a result.^5^, ^6^, ^7^

Based on the formula Force = Pressure × Area, it was expected that the force required to puncture the simulated parietal pleura using Kelly clamps would be double to triple that of the force required using the fine artery forceps. The median force measured to puncture the synthetic parietal pleura using the Kelly clamps was 52.91 N compared to the fine artery forceps 10.70 N. These results represent up to a fivefold increase in the force required when using the Kelly clamps. The variability in force using Kelly clamps may be a reflection of a loss of finesse using a single‐handed technique.

Newton's second law explains that the acceleration of an object produced by a net force is directly proportional to the magnitude of the net force, in the same direction as the net force. Given the increase in simulated pleural puncture force required when using Kelly clamps, there is an increased risk that the clamps may accelerate into the pleural cavity once the resistance from the pleural is removed following puncture, with an increased chance of piercing the lung.

This is well described by Begg (2014) ‘…At the moment of puncture when the tissue fails at the tip of the instrument, the reaction force applied to the device by the tissue drops suddenly and significantly but the force applied by the user remains until the user can react. This unbalanced force causes the device to accelerate suddenly into the patient… If this sudden acceleration is great enough, the physician's reaction time is sufficiently long, or the tissue has deformed far enough inwards prior to puncture, the tip of the device may travel farther than intended and impact underlying tissues or organs’.^1^

The study had several limitations. The instrument used was not blinded to the operator. Also, the study was performed on a mannequin by a single operator using a single‐handed technique. There may be significant variability in the force delivered among operators. A two‐handed technique using the non‐dominant hand to steady the longer Kelly clamps could potentially reduce uncontrolled acceleration and reduce the risk of the forceps puncturing the underlying lung.

This *in vitro* study using a simulated model did not adjust for *in vivo* variables such as chest wall depth, tissue compliance or ability of the clinician to safely define the tract for intercostal catheter insertion using smaller equipment. The authors use a skin incision of ≥3 cm to compensate for the latter. Also, the authors teach and practice a one‐handed technique for pleural puncture, and this technique reflects that practice. Alternative finger holds that may limit the risk of pulmonary injury were not explored. There are factors other than force that may impact pleural decompression safety. Both are worthy of further studies.

Nonetheless, the study findings are consistent with the expected engineering outcome. Both indicate that it may be safer to use fine artery forceps in preference to Kelly clamps when puncturing the pleural.^2^

## Conclusion

The present study demonstrated a significant reduction in the force required to puncture simulated parietal pleura when using fine artery forceps compared to when using Kelly clamps. The model did not adjust for *in vivo* operator and patient related variables, and these results could be further verified on live subjects requiring emergency pleural decompression. However, based on these data, it is argued that clinicians may reduce the risk of pulmonary injury by using fine artery forceps and not Kelly clamps when performing emergency pleural puncture.

## Data Availability

The data that support the findings of this study are available from the corresponding author upon reasonable request.
